# Raman chemometric urinalysis (Rametrix) as a screen for bladder cancer

**DOI:** 10.1371/journal.pone.0237070

**Published:** 2020-08-21

**Authors:** Herbert M. Huttanus, Tommy Vu, Georgi Guruli, Andrew Tracey, William Carswell, Neveen Said, Pang Du, Bing G. Parkinson, Giuseppe Orlando, John L. Robertson, Ryan S. Senger

**Affiliations:** 1 Department of Biological Systems Engineering, Virginia Tech, Blacksburg, Virginia, United States of America; 2 Department of Chemical Engineering, Virginia Tech, Blacksburg, Virginia, United States of America; 3 Department of Surgery–Urology, Virginia Commonwealth University, Richmond, Virginia, United States of America; 4 Department of Cancer Biology, Wake Forest School of Medicine, Winston-Salem, North Carolina, United States of America; 5 Department of Statistics, Virginia Tech, Blacksburg, Virginia, United States of America; 6 Internal Medicine, Lewis-Gale Medical Center, Salem, Virginia, United States of America; 7 Department of Surgical Sciences–Transplant, Wake Forest University Baptist Medical Center, Winston-Salem, North Carolina, United States of America; 8 DialySensors Inc., Blacksburg, Virginia, United States of America; 9 Department of Biomedical Engineering and Mechanics, Virginia Tech, Blacksburg, Virginia, United States of America; Chang Gung Memorial Hospital at Linkou, TAIWAN

## Abstract

Bladder cancer (BCA) is relatively common and potentially recurrent/progressive disease. It is also costly to detect, treat, and control. Definitive diagnosis is made by examination of urine sediment, imaging, direct visualization (cystoscopy), and invasive biopsy of suspect bladder lesions. There are currently no widely-used BCA-specific biomarker urine screening tests for early BCA or for following patients during/after therapy. Urine metabolomic screening for biomarkers is costly and generally unavailable for clinical use. In response, we developed Raman spectroscopy-based chemometric urinalysis (Rametrix™) as a direct liquid urine screening method for detecting complex molecular signatures in urine associated with BCA and other genitourinary tract pathologies. In particular, the Rametrix^TM^ screen used principal components (PCs) of urine Raman spectra to build discriminant analysis models that indicate the presence/absence of disease. The number of PCs included was varied, and all models were cross-validated by leave-one-out analysis. In Study 1 reported here, we tested the Rametrix™ screen using urine specimens from 56 consented patients from a urology clinic. This proof-of-concept study contained 17 urine specimens with active BCA (BCA-positive), 32 urine specimens from patients with other genitourinary tract pathologies, seven specimens from healthy patients, and the urinalysis control Surine^TM^. Using a model built with 22 PCs, BCA was detected with 80.4% accuracy, 82.4% sensitivity, 79.5% specificity, 63.6% positive predictive value (PPV), and 91.2% negative predictive value (NPV). Based on the number of PCs included, we found the Rametrix^TM^ screen could be fine-tuned for either high sensitivity or specificity. In other studies reported here, Rametrix^TM^ was also able to differentiate between urine specimens from patients with BCA and other genitourinary pathologies and those obtained from patients with end-stage kidney disease (ESKD). While larger studies are needed to improve Rametrix^TM^ models and demonstrate clinical relevance, this study demonstrates the ability of the Rametrix^TM^ screen to differentiate urine of BCA-positive patients. Molecular signature variances in the urine metabolome of BCA patients included changes in: phosphatidylinositol, nucleic acids, protein (particularly collagen), aromatic amino acids, and carotenoids.

## Introduction

Bladder cancer (BCA) is common and the most costly type of cancer to treat [1]. More than 80,000 new cases were expected to be diagnosed in 2019 (4.6% of all newly-diagnosed cancer cases), and almost 18,000 patients would die due to tumor progression and treatment failure [2]. There are currently over 577,400 patients under treatment [3]. The five-year survival rate for BCA is 77.4%, and early stage disease is correlated with better five-year survival (Stage 0–98%, Stage 1–88%, Stage 2–63%). Five-year survival is worse for more advanced stages (Stage 3–46%, Stage 4–15%). Approximately 30% of all new muscle-invasive cases are first diagnosed in Stages 2–4 [4].

Early detection of asymptomatic BCA is problematic. The onset of clinical symptoms (e.g., hematuria, dysuria, urgency, lower back pain) usually triggers further clinical and diagnostic investigation [5–7]. Routine urinalysis is not useful for early BCA detection, and the significance of minimal hematuria in specimens is debatable [8,9]. Definitive diagnosis of BCA in symptomatic patients is accomplished with a combination of imaging studies, urine cytology, direct bladder examination (i.e., cystoscopy) and tests for BCA biomarkers [10–16]. None of the current urine-based biomarker tests have gained wide acceptance or become a standard-of-care for screening or patient follow-up [17–19].

Several groups have investigated the use of urine metabolomic profiling for detection or clinical follow-up of BCA. Boutara and co-workers [20] used urine metabolomic profiling to characterize thousands of unique molecules in normal urine, and Shi et. al. [21] reviewed the current literature on BCA-associated metabolomic markers. Burton and Ma [22] reported alterations in metabolic pathways and the presence of pteridines, acylcarnitine derivatives, and nucleic acid metabolites in the urine of BCA patients. In related work, Kalim and Rhee [23], Hao et. al. [24], and Grams et. al. [25] used metabolomic profiling of blood and urine to detect renal disease and produced unique data that could be used to differentiate upper and lower urinary tract pathologies, such as BCA.

All current diagnostic procedures, including testing for urine-based biomarkers, are either (i) costly, (ii) require some degree of expertise to achieve valid results, (iii) invasive, (iv) not reliably sensitive or specific, (v) highly dependent on sample quality and stage of tumor growth, (vi) analytical resource intensive (e.g., requiring mass spectroscopy), and/or (vii) not scalable for mass screening. A simple, non-invasive and reliable screening technology for detection of BCA could reduce the use of invasive and costly evaluation tests for the patients unlikely to have cancer, and expedite the diagnosis and treatment for those who do. For BCA surveillance, such a test could improve the identification of the disease recurrence/progression, and reduce cystoscopy in patients with low risk.

Unlike some other tumors (e.g., prostate cancer), no case of BCA can be left untreated, since it will predictably become symptomatic and will progress without treatment. The earlier a tumor is diagnosed, the greater the chances are that BCA will be curable or controllable, or that less aggressive treatment can be used (i.e., bladder-sparing therapies). Thus, an accurate, non-invasive screening technology could be used clinically for annual urinalysis of the population at increased risk for developing BCA (e.g., users of tobacco products; >27 million people in the US) [7], for monitoring the efficacy of therapy in patients who have BCA (over 577,000 people), and monitoring patients for tumor recurrence/progression.

We have developed a novel screening technology, Raman chemometric urinalysis (Rametrix^TM^), for use in detecting BCA markers (multi-molecular signatures) in urine. Rametrix^TM^ is based on Raman spectroscopic analysis of urine and multivariate statistical analysis of the spectral data. Raman spectroscopy, itself, is a mature, well-studied, powerful technology that has been applied routinely to analyze the chemical composition of a wide variety of solid, liquid, and biological samples [26–33]. For analysis, a sample is excited by a monochromatic laser, and the resulting spectrum shows the intensity of Raman scattered radiation (arising from chemical bond rotations, stretching, and bending) as a function of frequency [34]. When applied to the analysis of urine, Raman spectroscopy has the following advantages: (i) it is label-free and requires minimal sample preparation, (ii) chemical composition data is returned in near/real-time, (iii) there is minimal spectral interference from water (unlike infrared methods), (iv) scanning through glass is possible, (v) it is non-destructive to the sample, and (vi) it can be easily scaled for large numbers of samples (through automation). These advantages make Raman spectroscopy an attractive method for screening urine for the detection of BCA and other urinary tract pathologies.

Urine contains hundreds of individual molecules that reflect metabolism and health, as well as the important physiologic activities of the urinary system [20]. The composition is highly variable among individuals and, in fact, varies substantially in every individual, every day, depending on activity, diet, metabolism, ingestion of exogenous drugs and chemicals, state of hydration, and renal function. In a single Raman spectral scan (obtained in < 5 minutes), many of these molecules can be identified easily and reliably as spectral bands that can be correlated to analytical standards in Raman reference libraries. For example, small molecules (e.g., urea, creatinine, uric acid, glucose) and macromolecules commonly used to assess health and renal function all produce Raman intensity bands at specific wave numbers. These *collectively* create a unique spectral “fingerprint” of a urine specimen’s molecular composition. Understandably, the presence of BCA and other bladder pathologies has a profound effect on urine composition. These compositional changes are present in the spectral fingerprints, and they can be resolved by multivariate statistical models in Rametrix^TM^ [29,35].

Others have recognized the value of using Raman spectroscopy for studying urinary tract disease, including BCA. DeJong and coworkers [36] were able to distinguish a unique Raman signature associated with BCA cells in surgical (excisional) biopsy touch preparations. Bird and co-workers [37] used IR Raman microscopy for detection of BCA in cytology preparations. Canetta and co-workers [38] used modulated Raman spectroscopy to differentiate unique Raman spectral signatures in preparations of urothelial and bladder cancer cells derived from tissue cultures. Shapiro and co-workers [39] used Raman micro-spectroscopy to evaluate bladder cancer lesions and found a unique spectral band at wavelength 1,584 cm^-1^ that distinguished tumor vs. non-tumor tissue and low-grade vs. high-grade BCA. Kerr and co-workers [40] used Raman micro-spectroscopy to differentiate bladder cancer cells from other urine sediments. Yang et al. [41] created a surface enhanced Raman scattering (SERS) assay for specific receptors on cancer cells. The signal amplification properties of SERS allow these molecules to be illuminated in liquid urine specimens. Further, Raman spectroscopy-based screens with Fe_3_O_4_ functionalized surfaces have been developed for detecting urine crystals, which may go on to form stones [42, 43].

As can be seen, a large percentage of the published literature on the use of Raman spectroscopy for detection of BCA is centered on the evaluation of cytologic preparations, with a small fraction focused on evaluation of the urine metabolome. Our Rametrix^TM^ technology, uniquely, relies on discerning molecular changes in liquid urine, making it significantly more practical and suitable for mass screening of specimens. Here, we report the results of a preliminary clinical study where Raman spectroscopy was used with the Rametrix™ LITE [29] and Rametrix^TM^ PRO [44] spectral processing methods to analyze urine specimens from BCA patients and from patients with other genitourinary diseases. We performed the study to test the hypothesis that “unique Raman spectral patterns (i.e., molecular fingerprints) are associated with BCA and can be detected in liquid urine by Rametrix^TM^.” We compared the results of these Raman spectroscopic analyses with those obtained from Rametrix™ analysis of urine from healthy volunteers [35], those with end-stage kidney disease (ESKD) [45], and a synthetic urinalysis analytical standard (Surine^TM^). We also evaluated Rametrix^TM^ as a screen for BCA by calculating its sensitivity, specificity, positive predictive value (PPV), and negative predictive value (NPV) metrics. These metrics are critical to assessing the potential usefulness of the screening test [46], as high sensitivity (true-positive screen result) leads to a higher percentage of BCA cases being identified, and high specificity (true-negative screen result) minimizes false-positive screen results that can lead to unnecessary (and invasive) tests and examinations [47].

## Materials and methods

### Ethics statement

This study was approved by Virginia Commonwealth University IRB #HM20006879; Virginia Tech IRB #VT-IRB 15–703; and Frenova research agreement RPP/177151.2 (Fresenius Renal Research; 920 Winter Street, Waltham, MA 02451). Informed written consent was obtained for collection of urine specimens from all subjects in this study. Specimens were collected from (i) patients and volunteers presenting at a urology clinic at a large tertiary-care medical center, between September 2016 and April 2017; (ii) healthy volunteers affiliated with Virginia Tech; and (iii) patients undergoing peritoneal dialysis therapies for ESKD. At the time of collection, specimens were de-identified and assigned a code.

### Urine specimen preparation

Urine specimens were collected following IRB approval and obtaining patient written consent; specifics are provided below for each group of patients. Specimens were stored at -35°C for no more than four weeks prior to analysis; such conditions preserve the veracity of the Raman signature (see below Specimen Stability Validation). Stored specimens were thawed for approximately 25 minutes in an incubator at 37°C in preparation for analysis. A total of 1.5 mL of each urine specimen was then aliquoted into glass vials, which were then sealed. Specimens exhibiting precipitates were vortexed briefly to re-suspend and dissolve these prior to Raman scanning. Surine^TM^ (Dyna-Tek Industries, Lenexa, KS), a synthetic analytical standard, was used as the urinalysis control and was also prepared in a similar manner.

### Raman spectroscopy

Urine specimens were analyzed as bulk liquids using a PeakSeeker^TM^ dispersive Raman spectrometer (Agiltron; Woburn, MA). Specimens were each scanned 10 times using 785 nm 100 mW laser intensity, with 15 second exposure time and a 15 second delay between each scan. Specimens were scanned in a random order, and Surine^TM^ was scanned with each batch of urine specimens analyzed.

### Urology clinic patients dataset

Urine specimens were obtained from 56 subjects (patients and volunteers) presenting at a urology clinic, as described above. The sample size of this study was determined by the collection period duration. The characteristics of the study population are presented in Table 1. Mid-stream free-catch urine specimens were acquired in sterile sample cups from the study subjects. Specimen integrity was preserved prior to Raman scanning using methods described above. Patient diagnosis was noted during collection so that results could be correlated with analytical data.

**Table 1:** Characteristics of study populations and categories of genitourinary tract pathology studied.

Dataset	Total Number of Specimens	Number of Males	Number of Females	Median Age (Years)
Urology Clinic Patients Dataset	56	35	21	62
*BCA patients (active)*	17	8	9	70
*BCA patients (inactive a/o under treatment)*	8	7	1	62
*Genitourinary cancer (Renal*, *prostate)*	8	7	1	60.5
*Other non-neoplastic genitourinary (GU) disease*	16	11	5	59
*Urology clinic healthy volunteers*	7	2	5	40
Healthy Volunteers Dataset	56	13	43	22
Nephrology Clinic Patients Dataset	56	N/A	N/A	N/A

A definitive diagnosis for each patient presenting with clinical signs indicative of BCA was made using a combination of patient history, standard clinical pathology laboratory studies, imaging, direct visualization (cystoscopy) and a confirmatory biopsy. The definitive diagnosis was used to classify urine specimens for subsequent Rametrix^TM^ analysis. Clinical diagnoses for other patients were made using a combination of patient history, standard clinical pathology laboratory studies, imaging, direct visualization (cystoscopy) and confirmatory biopsy, as needed. The definitive diagnosis is referred to as the “Gold Standard” test when describing metrics to evaluate Rametrix^TM^ as a screening test. These metrics are defined later.

### Healthy volunteers dataset

For the study reference population, a subset of a previously published dataset [35] of consented healthy female and male volunteers, ranging in age from 19–69 years old (median age 22 years old) was used (Table 1). The state of “healthy” was defined as free of infectious or degenerative disease at the time of sample collection, and with no history/evidence of renal disease (based on laboratory measurements). Samples from healthy volunteers were handled in an identical manner to those described above.

### Nephrology clinic patient dataset

In previous research [45], urine specimens were collected from patients undergoing peritoneal dialysis therapies for ESKD. A subset of this larger patient database was used for this study (Table 1), and the data derived from many of these specimens have also been used with Rametrix^TM^ in other studies [29,44].

### Specimen stability validation

In unpublished research [48], the effects of storage conditions on urine specimens and Surine^TM^ molecular composition were studied in detail. Here, we present a subset of this data to validate storage at -35°C for four weeks. Surine^TM^ and two of the urine specimens used in this study were transferred to glass vials and were analyzed by Raman spectroscopy initially (t = 0), after an initial freeze/thaw, and then once every seven days for 12 weeks. All vials were thawed and vortexed briefly before analysis. Data were analyzed by Rametrix^TM^ LITE and statistical models as described below.

### Rametrix^TM^ analysis

Urine sample spectra were processed and analyzed using the Rametrix^TM^ LITE v1.1 [29] and PRO v1.0 [44] Toolboxes and the Statistics and Machine Learning Toolboxes in MATLAB r2017b (The MathWorks, Inc.; Natick, MA). The Rametrix^TM^ LITE Toolbox was used for spectral processing and for building Principal Component Analysis (PCA) and Discriminant Analysis of Principal Components (DAPC) models. The Rametrix^TM^ PRO Toolbox was used to test the ability of DAPC models to classify “unknown” urine specimens by leave-one-out analysis. Statistical analyses including one-way Analysis of Variance (ANOVA) and pairwise comparisons, using Tukey’s Honestly Significant Difference (HSD), were performed in MATLAB.

#### Rametrix^TM^ LITE

For the PCA and DAPC model-building process with Rametrix^TM^ LITE, each Raman spectrum was assigned a classification based on the patient diagnosis (e.g., BCA-positive or BCA-negative). Each Raman spectrum was truncated to 400–1,800 cm^-1^, followed by baseline correction using the Goldindec algorithm [49] with the parameters:: baseline polynomial order = 3; estimated peak ratio = 0.5; smoothing window size = 5. All spectra from each urine specimen were then vector normalized and averaged. Next, PCA was applied with the Rametrix^TM^ LITE Toolbox, and a specified number of PCs was used to create DAPC model(s). The Rametrix^TM^ LITE Toolbox also automated calculation of Raman shift contributions to PCA and DAPC models. In this procedure, the contribution of each Raman shift to the separation of classification groups (e.g., BCA-positive vs. BCA-negative) was determined. Those Raman shifts with significantly large contributions were investigated further through the use of Raman spectral libraries. This enabled conversion of spectral signatures into inferences about the metabolome of BCA-positive urine. Finally, ANOVA and pairwise comparisons were performed in MATLAB following Rametrix^TM^ LITE calculations.

#### Rametrix^TM^ PRO

**Fig 1:**
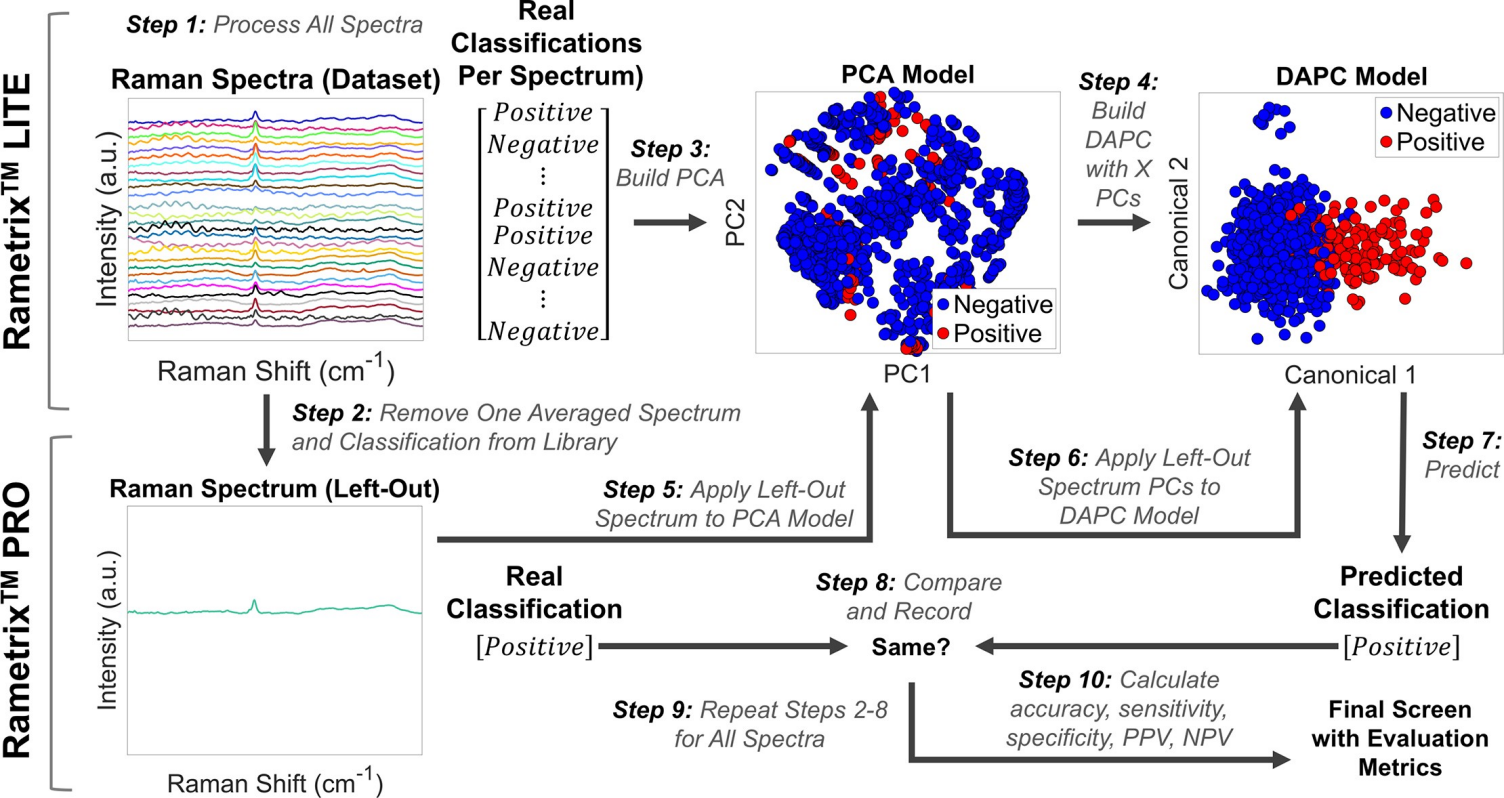
Flow diagram of Rametrix^TM^ calculations and the leave-one-out routine. Rametrix^TM^ LITE comprises steps 1, 3, and 4. Rametrix^TM^ PRO comprises steps 2, 5–10.

The Rametrix^TM^-based urine screen for BCA involves obtaining a Raman spectrum of urine, processing the spectrum as described above, reducing the spectrum to its PCs, and using those with the DAPC model to generate a prediction (e.g., BCA-positive or BCA-negative). To do this, Rametrix^TM^ PRO tested DAPC models for their predictive capabilities following their construction with Rametrix^TM^ LITE. Specifically, Rametrix^TM^ PRO performed a leave-one-out analysis over all models and datasets. This procedure is shown as a flow-diagram in Fig 1 Leave-one-out analysis is a subset of K-fold analysis [50] and ensures every urine specimen in the dataset is evaluated as an “unknown” at some point in the routine (Fig 1). In particular, the leave-one-out analysis removed one spectrum from the dataset (or spectral library) and treated it as an unknown. PCA and DAPC models were constructed using the remaining spectra, and the classification of the unknown was predicted by the model (e.g., “BCA-positive” or “BCA-negative”). This process was repeated for each spectrum in the dataset. With correct/incorrect predictions for every spectrum in the dataset, the urine screen evaluation metrics were calculated as described below. In addition, this process was repeated for every study presented in the Results section.

#### Evaluation metrics

Urine screen accuracy (i.e., adequacy) was calculated as the percentage of spectra classifications predicted correctly in the leave-one-out routines. Sensitivity, specificity, PPV, and NPV evaluation metrics were calculated as described in the literature [46]. Briefly, sensitivity is the true-positive rate (reported as percentage) of the Rametrix^TM^ screen. This is the proportion of BCA cases confirmed via Gold Standard test (i.e., the definitive diagnosis described earlier) which also test positive according to the screen. The specificity is the true-negative rate and is the proportion of negative cases confirmed via Gold Standard which also test negative by the screening test. The PPV indicates the proportion of positive screening tests that then test positive via Gold Standard. The NPV indicates the proportion of negative screening tests that then test negative via Gold Standard [46].

These metrics (i.e., accuracy, sensitivity, specificity, PPV, and NPV) were compared to their random chance rates. For example, for the BCA-positive or BCA-negative classification, the random chance rates of all metrics were all 50%.

#### DAPC models for high sensitivity and specificity

Different numbers of PCs were used to construct DAPC models. This frequently impacted model performance and resulted in different metric values. Thus, results from multiple models are reported for each scenario tested in this study. In all cases, at least one high-sensitivity and one high-specificity model are reported with associated values of all other metrics (i.e., accuracy, PPV, and NPV).

#### ANOVA and pairwise comparisons

The classification groups (e.g., BCA-positive and BCA-negative) were also tested for statistically significant differences among their spectra using ANOVA and pairwise comparisons using Tukey’s HSD procedure of Total Principal Component Distance (TPD). To do this, each spectrum was reduced from several hundred of Raman intensity values (one at each wavenumber) into a single numerical value. This was done by calculating the distance between the top four PCs of each spectrum and a reference spectrum, as shown in Eq 1. In this research, the spectrum for Surine^TM^ served as the reference. The TPD calculation has been used in other analyses with Rametrix^TM^, and more details have been published elsewhere [35]. In Eq 1, *P_u,i_* is the value of the *i^th^* PC of urine spectrum *u*, and *P_ref,i_* is the value of the *i^th^* PC of the reference (i.e., Surine^TM^) spectrum. In contrast to Rametrix^TM^ PRO analysis of DAPC models, Rametrix^TM^ LITE and statistical analyses were performed without averaging replicate spectra for each urine specimen.

$$TPD=\sum_{i=1}^{4}\sqrt{{\left(P_{u,i}-P_{ref,i}\right)}^{2}}$$ Eq 1

### Defining the studies and goals

Two preliminary studies were conducted followed by five larger studies involving patient datasets described in Table 1. In the first preliminary study, the storage conditions of the urine specimens and Surine^TM^ were validated. In the second preliminary study, the Raman spectra were inspected visually to determine if chemometric methods by Rametrix^TM^ were needed in this study. The larger patient studies are listed in Table 2. In Study 1, the 56 urine specimens of the urology clinic dataset were used. Those (17) with active BCA were classified as “BCA-positive.” The remaining specimens were classified as “BCA-negative.” This study was designed to determine if urine from patients with active BCA could be distinguished from urine of patients (39) from the same urology clinic who did not (classified as “BCA-negative”). In Study 2, the 56 urine specimens from the Healthy Volunteers dataset were added as additional “BCA-negative” specimens to the dataset described for Study 1. Here, the goal was to determine if adding urine spectra from healthy volunteers (median age of 22 years) would skew the results obtained in Study 1 (median age of 62 years). In Study 3, the Nephrology Clinic dataset, composed of urine from ESKD patients, was added to the “BCA-negative” classification. ESKD is known to affect urine molecular composition and Raman spectral characteristics [45]. We then cross-referenced these spectral differences with a Raman band database [30] to identify potential molecules significantly altered in BCA-positive urine. We refer to this as the “molecular signature” of BCA based on our Rametrix^TM^ urine screen. In Study 4, we sought to determine whether the urine specimens could be distinguished by clinic type. The goal was to identify specific urine spectral signatures of patients visiting urology and nephrology clinics, independent of patient health status, and whether these signatures can be distinguished from urine of healthy volunteers. Finally, in Study 5, the Urology Clinic dataset was used, and specimens were re-classified as “Genitourinary (GU) Cancer,” “Other GU Disease,” and “Healthy.” The goal was to differentiate among all of these to determine if a disease type could be identified among urology clinic patients.

**Table 2:** Definition of studies and urine specimen classifications.

Study	Datasets	Classifications
Study 1	Urology Clinic Patients + Surine^TM^	BCA-Positive, BCA-Negative, Surine^TM^
Study 2	Urology Clinic Patients + Healthy Volunteers + Surine^TM^	BCA-Positive, BCA-Negative, Surine^TM^
Study 3	Urology Clinic Patients + Healthy Volunteers + Nephrology Clinic Patients + Surine^TM^	BCA-Positive, BCA-Negative, Surine^TM^
Study 4	Urology Clinic Patients + Healthy Volunteers + Nephrology Clinic Patients + Surine^TM^	Urology Clinic Patients, Nephrology Clinic Patients, Healthy Volunteers, Surine^TM^
Study 5	Urology Clinic Patients + 9 Healthy Volunteers + Surine^TM^	GU Cancer, Other GU Disease, Healthy, Surine^TM^

### Public access

The raw TPD values, statistical analyses, and Rametrix^TM^ PRO results are included in the S1 File. The copyrighted raw Raman spectra data of the Urology Clinic dataset used in this study as well as the Rametrix^TM^ LITE and PRO Toolboxes are offered under license agreement through GitHub. Relevant links are as follows:

- Raman spectra files: https://github.com/SengerLab/Raman-Scans/tree/BCA
- Rametrix^TM^ LITE: https://github.com/SengerLab/RametrixLITEToolbox
- Rametrix^TM^ PRO: https://github.com/SengerLab/RametrixPROToolbox

## Results

### Stability validation

Surine^TM^ and two urine specimens used in this study were stored in triplicate vials at -35°C and analyzed weekly for 12 weeks. The purpose of the study was to justify storage of all urine specimens used in the study for up to four weeks at -35°C. Raw Raman spectra files are available through GitHub (see Methods), and statistical results are given in the S1 File. The spectra were analyzed, first, with Rametrix^TM^ LITE by averaging the 10 Raman scans per analysis for each vial, and truncating, baselining, and vector normalizing, as described in the Methods section. PCA, with respect to storage time, was applied with Rametrix^TM^ LITE, followed by calculation of TPD (Eq 1). Here, the initial time point (time = 0) served as the reference (*P_ref_*) in Eq 1, and the TPD values were analyzed by ANOVA and pairwise comparisons. ANOVA results revealed no statistical significance of storage time (p = 0.29). Pairwise comparisons allowed each time point to be compared with the initial time point. Here, all p-values were greater than 0.54, and those of the first four weeks of storage were all greater than 0.91. These results coordinate well with our larger study [48], suggesting urine specimens can be stored at -35°C for at least four weeks while awaiting analysis.

### Identifying BCA through direct comparisons of Raman spectra

**Fig 2:**
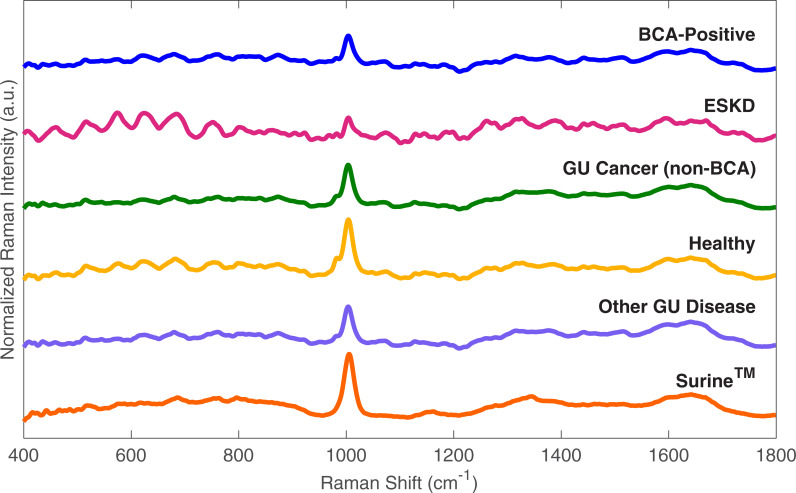
Representative baselined and vector normalized urine spectra. BCA-positive is representative of patients with active BCA. GU Cancer (non-BCA) includes genitourinary cancers (Renal, prostate). Other GU Disease includes those patients with non-neoplastic diseases.

Raman spectral data from the Urology Clinic Patients dataset were processed and vector normalized using the Rametrix^TM^ LITE Toolbox for MATLAB. Representative spectra are shown in Fig 2 collected from analysis of urine from patients with a BCA diagnosis, ESKD, non-BCA GU cancer (e.g., renal and prostate), healthy volunteers and patients, other non-cancer GU diseases, and Surine^TM^. There are visible differences between each of the spectra, with some, especially for ESKD, appearing pronounced. Urine spectra obtained from urology clinic patients, which included BCA-positive, healthy patients, non-BCA GU cancer, and non-cancer GU diseases revealed no large defining spectral characteristics of BCA upon visual inspection, which is consistent with the current lack of urine biomarkers. This also indicates computational analyses are needed to detect and quantify differences. Therefore, PCA, DAPC, and multivariate statistical analyses, including ANOVA and pairwise comparisons, were performed.

With these methods, it was determined whether defining characteristics of BCA existed in the Raman spectra. The non-BCA GU Cancer, healthy patients, and non-cancer GU diseases spectra were combined to form a “BCA-negative” classification and were compared against the BCA-positive spectra. The TPD of each sample, relative to a Surine^TM^ control, was calculated as described above (Eq 1); the urine specimens from BCA-positive patients were found to be significantly different from BCA-negative patients in the dataset by both ANOVA and pairwise comparisons (*p* < 0.001). Raw TPD values and statistical analysis results are available in the S1 File. This prompted further analyses to develop a Rametrix^TM^-based urine screen for BCA and discover the urine metabolome differences of BCA-positive patients.

### Study 1: Identifying active BCA in the urology clinic patients dataset

#### Rametrix^TM^ LITE results

**Fig 3:**
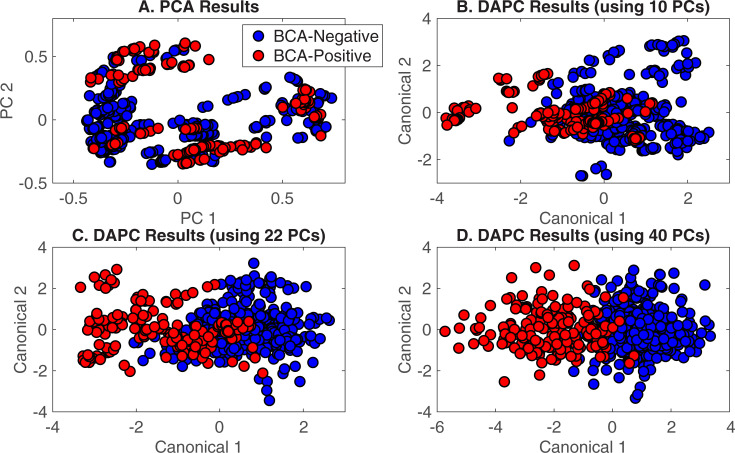
Rametrix^TM^ LITE results for BCA-positive and BCA-negative spectra. A) PCA results, B) DAPC results (model built with 10 PCs), C) DAPC results (model built with 22 PCs), D) DAPC results (model built with 40 PCs).

In the initial analysis, differences between urine from BCA-positive and BCA-negative patients from the Urology Clinic dataset (Table 1) were explored. To build a predictive DAPC model, spectra from the Urology Clinic patient dataset were classified simply as “BCA-positive” (urine from a patient with active BCA) and “BCA-negative” (urine from a urology clinic patient without active BCA), as shown in Table 2.) PCA and DAPC analyses were performed with Rametrix^TM^ LITE. Plots shown in Fig 3 provide a visual representation of the statistical similarities/differences represented by clustering. Here, each data point of Fig 3 represents one Raman spectrum. Each PC represents a direction of variation among the different spectra, with the first PC being the direction of greatest variation, the second PC being the direction of next greatest variation, and so forth. A total of *n* minus *1* PCs were used, with *n* being the total number of spectra. The PCA plot (Fig 3A) does not have outliers or data points indicative of system errors, but the data do not cluster according by spectrum classification. Thus, DAPC was needed to find spectral differences according to classification and build a model capable of identifying an unknown specimen correctly.

DAPC results (Fig 3B–3D) show specific clustering of the data classes after variance between groups is factored into the analysis. Unlike PCA, DAPC requires samples to be grouped prior to analysis and uses that classification information to generate canonicals, with each canonical representing (e. g., defined as) an axis of variation between the different classes. The DAPC plots (Fig 3B–3D) show each Raman spectrum condensed into a single data point on the plot, with the first two (of several) canonicals represented on the *x-* and *y-*axes. By accounting for the variation between dataset classes, DAPC plots tend to show more distinct clustering than PCA. Thus, the role of PCA in Rametrix^TM^ is to reduce a Raman spectrum from 1,400 data points (intensity values over the 400–1,800 cm^-1^ Raman shift) to a smaller number of PCs that can be used in DAPC. In this case (Fig 3B–3D), the specimens from BCA-positive patients (red) clustered away from specimens from BCA-negative individuals (blue) along canonical 1, and clustering improved as more PCs were included in the DAPC model. Thus, these models were built using the top 10, 22, and 40 of the 552 PCs generated by PCA. These represented up to 99.6% of the dataset variance. While using a large number of PCs can lead to effective clustering in DAPC plots (Fig 3D), using too many PCs can result in “over-fitting” the data. This results in poor performance when classifying unknown specimens. This was evaluated with leave-one-out predictions using Rametrix^TM^ PRO.

#### Evaluating the DAPC model with Rametrix^TM^ PRO

**Fig 4:**
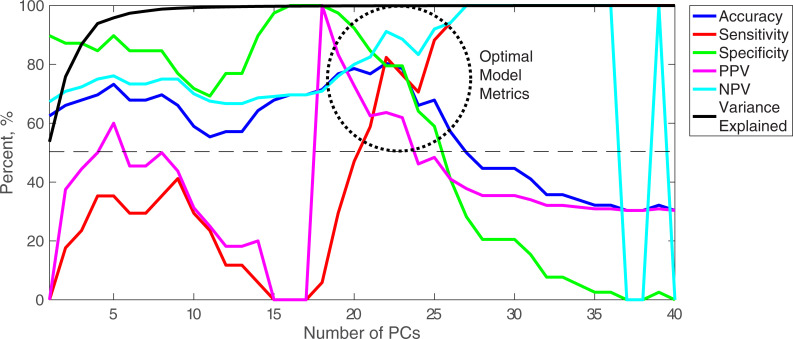
Rametrix^TM^ PRO results for BCA-positive and BCA-negative spectra. Accuracy, sensitivity, specificity, PPV, and NPV results are given for leave-one-out analyses. The analysis was repeated for several DAPC models constructed with numbers of PCs. The variance explained refers to the dataset variance explained by the number of PCs included in the DAPC model.

The leave-one-out analysis was carried out using Rametrix^TM^ PRO on DAPC models built with up to 40 PCs. The output includes the evaluation metrics (accuracy, sensitivity, specificity, PPV, and NPV), the number of PCs used to build the DAPC model, and the percent of dataset variance explained by those PCs. Results are presented graphically as Fig 4, highlighted metric values are given in Table 3, and all metric values are given in the S1 File. For the Urology Clinic Patients dataset, models constructed with a low number (< 20) of PCs exhibited low sensitivity (< 50%) and PPV (< 60%) metrics but specificity values that approached 90% and NPVs above 75%. As the number of PCs increased, sensitivity and PPV increased non-linearly while specificity and NPV decreased. Between 19 and 23 PCs (region circled on Fig 4 and metric values given in Table 3), accuracy was maximized at about 80%, but different values of sensitivity, specificity, PPV, and NPV were observed. This suggests Rametrix^TM^ could be operated at high accuracy but be fine-tuned to favor any one (or multiple) metric(s). For example, the sensitivity range was 29.4 to 82.4% over the five models highlighted. Specificity showed an inverse relationship, relative to sensitivity, with the maximum being 97.4%. The model built with 22 PCs showed the highest accuracy (80.4%) with relatively high values of the other metrics, and all metrics were above the 50% random chance value.

**Table 3:** DAPC model metrics for BCA-positive and BCA-negative spectra of the Urology clinic patients dataset*.

PCs	Accuracy	Sensitivity	Specificity	PPV	NPV
19	77.7%	29.4%	97.4%	83.3%	76.0%
20	78.6%	47.1%	92.3%	72.7%	80.0%
21	76.8%	58.8%	84.6%	62.5%	82.5%
22	80.4%	82.4%	79.5%	63.6%	91.2%
23	78.6%	76.5%	79.5%	61.9%	88.6%

* The BCA-positive/BCA-negative ratio of the Urology Clinic Patients dataset was approximately 30/70%.

### Studies 2 and 3: Adding spectra from healthy volunteers and ESKD patients to the dataset

**Fig 5:**
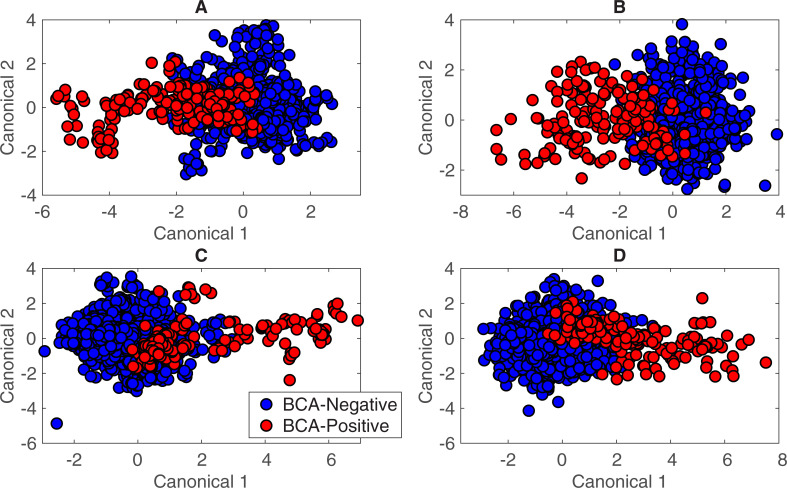
DAPC model results for BCA-positive and BCA-negative spectra when including healthy volunteers and ESKD patients. A) Urology clinic patients and healthy volunteers (26 PCs), B) Urology clinic patients and healthy volunteers (50 PCs), C) Urology clinic patients, healthy volunteers, and nephrology clinic patients (ESKD) (22 PCs), D) Urology clinic patients, healthy volunteers, and nephrology clinic patients (ESKD) (50 PCs). DAPC models were built with the number of PCs listed.

The BCA prediction model was re-built using an expanded dataset containing more BCA-negative samples. By adding 56 healthy volunteers from the VT dataset [35], pairwise comparisons of TPD data continued to show significant differences between BCA-positive and BCA-negative spectra (*p* < 0.001). DAPC results from models built from this expanded dataset with 26 and 50 PCs are shown in Fig 5A and 5B. Then, urine spectra from 56 late-stage ESKD patients (selected randomly from our larger dataset) were added to the dataset to generate the DAPC plots using 22 and 50 PCs, respectively, in Fig 5C and 5D. Despite having a condition known to alter their urine Raman spectra [45], the spectra from ESKD patient specimens were clearly different from spectra of BCA-positive patients and clustered with other BCA-negative individuals. The spectra were still distinguishable between BCA-positive and BCA-negative by TPD calculations and pairwise comparisons (*p* < 0.001).

Highlighted Rametrix^TM^ PRO results from analysis of these datasets are given in Table 4, and all metrics models built with one through fifty (1–50) PCs are given in the S1 File. When the Healthy Volunteers dataset was added to the Urology Clinic Patients database, the percentage of BCA-positive patients dropped from 30% of the total spectra to 15%. This influenced model metrics, as seen by comparing Tables 3 and 4. Highly specific models were generated (i.e., specificity reaching 100%), but this came at the expense of sensitivity and PPV. For example, the model in Table 4 built with 19 PCs had one of the higher PPV metrics (66.7%) for a comparatively high value of overall accuracy (86.6%). This means that for every positive screen, two out of three patients would test positive with Gold Standard testing. In addition, only 23.5% of positive cases (according to the Gold Standard) would test positive with the screening test, which is a measure of the screening test sensitivity. In addition, a model was found with 26 PCs that yielded high accuracy (83.9%), sensitivity (58.8%), and specificity (88.4%), but the PPV showed that of those testing positive with the screen, only one of two would test positive by the Gold Standard. This study shows the value of maintaining a relatively balanced dataset between the number of BCA-positive and BCA-negative cases.

**Table 4:** DAPC model metrics for BCA-positive and BCA-negative spectra of Urology clinic patients, healthy volunteers, and nephrology clinic patients.

Datasets*	PCs	Accuracy	Sensitivity	Specificity	PPV	NPV
Urology Clinic Patients + Healthy Volunteers	19	86.6%	23.5%	97.9%	66.7%	87.7%
	26	83.9%	58.8%	88.4%	47.6%	92.3%
	35	58.0%	88.2%	52.6%	25.0%	96.2%
Urology Clinic Patients + Healthy Volunteers + Nephrology Clinic Patients	19	90.5%	11.8%	99.3%	66.7%	90.9%
	22	89.9%	23.5%	97.4%	50.0%	91.9%
	30	81.0%	58.8%	83.4%	28.6%	94.7%

* The values for the Urology Clinic Patients only are given in Table 2.

### Raman molecular signature of BCA

The Rametrix^TM^ LITE Toolbox was used to extract the Raman shift contributions to separation of the BCA-positive and BCA-negative spectra in the study involving all three datasets (Urology Clinic Patients, Healthy Volunteers, and Nephrology Clinic Patients). The DAPC model constructed with 30 PCs (Table 4) was used here. Several Raman shift contributions were observed for each model, and plots of these are given in the S1 File. The major Raman shift contributions in the top four PCs of PCA and the first four canonicals of DAPC were defined as those surpassing 0.3% contribution in S1A,S1B Fig in the S1 File. The molecular assignment of these bands was extracted from a reference Raman band database for biological molecules [30]. For PCA, these molecular assignments included: phosphatidylinositol (576 cm^-1^), nucleic acids (721, 827, and 1340 cm^-1^), protein (particularly collagen) (817, 981, 1065, 1127, and 1340 cm^-1^), and aromatic amino acids (827 and 1004 cm^-1^). For DAPC, all of these molecules were in agreement; although some Raman shifts differed. Additional assignments for DAPC included: cholesterol and fatty acids (702 and 1297 cm^-1^), monosaccharides (846 cm^-1^), glycogen (1048 cm^-1^), and carotenoids (1417 and 1518 cm^-1^). These are identified as the major components of the molecular signature for BCA; although, the direction (increase/decrease) and levels indicative of disease have not been established. We also note that several minor, still unidentified, components are present in this molecular signature for BCA (see S1A, S1B Fig in the S1 File).

### Study 4: Detecting differences by clinic type

**Fig 6:**
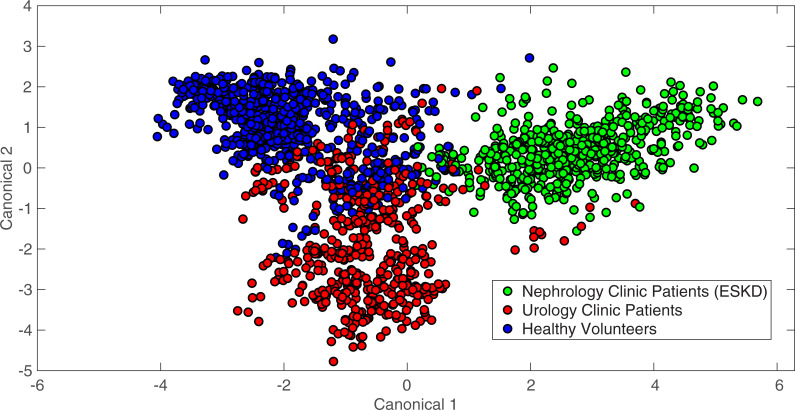
DAPC model results in clustering spectra by clinic type. Spectra were classified as Urology Clinic Patients, Healthy Volunteers, or Nephrology Clinic Patients. The DAPC model shown was built using 28 PCs.

DAPC models were also constructed to classify specimens as belonging to the broader classifications “Urology Clinic Patients,” “Healthy Volunteers,” or “Nephrology Clinic Patients.” For the purposes of this comparison, the healthy controls from the Urology Clinic Patient dataset were re-classified with the Healthy Volunteers. In this analysis, the Urology Clinic Patients classification included BCA-positive patients as well as those specified in Table 1. The Nephrology Clinic Patients were all being treated for ESKD with peritoneal dialysis at the time of urine specimen collection and analysis. DAPC results for a model built with 28 PCs are shown in Fig 6 and exhibited separation and clustering of these classes. Pairwise comparisons of TPD data (contained in the S1 File) showed both the Urology Clinic Patients and the Nephrology Clinic Patients were statistically different from the Healthy Volunteers classification and Surine^TM^ (*p* < 0.001), but they were not statistically different from one other (*p* = 0.92). It is expected that BCA, ESKD, and other GU pathologies all deviate from Surine^TM^ in different ways. In addition, it is possible that several of the Urology Clinic Patients may also have undiagnosed or underlying kidney disease, leading these to be identified as not statistically different according to TPD data. However, the clustering in Fig 6 suggests DAPC models may be able to discern among these patient types. Leave-one-out analysis was performed with Rametrix^TM^ PRO for each classification identified above, with the other two categories being considered negative results. Results of highlighted DAPC models are given in Table 5. Here, high-sensitivity and high-specificity model results (relative to all results) are given for each classification type. The full list of leave-one-out and statistical analysis results are included in the S1 File.

**Table 5:** DAPC model metrics for patient-type classifications.

Clinic Type	PCs	Accuracy	Sensitivity	Specificity	PPV	NPV
Urology Clinic Patients	10	84.5%	61.2%	94.1%	81.1%	85.5%
	20	85.7%	59.2%	96.6%	87.9%	85.2%
Nephrology Clinic Patients	15	92.9%	94.6%	92.0%	85.5%	15
	28	97.0%	91.1%	100.0%	100.0%	95.7%
Healthy Volunteers	21	90.5%	84.1%	94.3%	89.8%	90.8%
	35	88.1%	73.0%	97.1%	93.9%	85.7%

### Study 5: Detecting other cancer types and GU conditions

The ability of Rametrix^TM^ to detect BCA was broadened to detecting GU cancers (e.g., kidney or prostate) and other conditions identified in the Urology Clinic Patients dataset. It was also tested to see if differences could be detected between cancerous and non-cancerous GU pathologies. Towards this aim, we sorted the Urology Clinic Patients dataset into three new classifications: “GU Cancer,” “Other GU Disease,” and “Healthy.” To balance the relative sample abundance for each category, nine samples from the Healthy Volunteers dataset were added to the “Healthy” class of the Urology Clinic Patients dataset. Pairwise comparisons of TPD data showed GU Cancer spectra were statistically different from all others (i.e., Other GU Disease, Healthy, and Surine^TM^) (all *p* < 0.001). Interestingly, the only classifications with TPD values not statistically different from one another were Healthy and Other GU Disease (*p* = 0.99). This may point to limitations of Rametrix^TM^ to detect other forms of disease, or it may indicate a limitation of the current dataset, perhaps associated with the number of samples of each type that were analyzed.

Leave-one-out analysis was performed for each class individually. Highlighted leave-one-out results are given in Table 6, and all results are available in the S1 File. Multiple model results are given for each classification in Table 6, and these represent relatively high-sensitivity and high-specificity models for each classification. Model metrics showed to be lower than those of the other studies, pointing to additional challenges of resolving different pathologies from specimens represented in the Urology Patients Clinic dataset.

**Table 6:** DAPC model metrics for detecting GU pathologies.

GU Pathology	PCs	Accuracy	Sensitivity	Specificity	PPV	NPV
GU Cancer	30	66.15%	56.00%	72.50%	56.00%	72.50%
	33	58.46%	84.00%	42.50%	47.73%	80.95%
Other GU Disease	19	73.85%	18.75%	91.84%	42.86%	77.59%
	23	66.15%	62.50%	67.35%	38.46%	84.62%
Healthy	27	72.31%	45.83%	87.80%	68.75%	73.47%
	29	67.69%	66.67%	68.29%	55.17%	77.78%

## Discussion

The molecular composition of urine from BCA-positive patients differs from that of normal urine, and this is detectable by Raman spectroscopy and Rametrix^TM^. Additionally, the Raman spectra of BCA urine was found to also be different from urine of patients with ESKD [35,45] and other GU conditions. We were able to use Raman data and Rametrix^TM^ calculations to identify spectral characteristics unique to BCA-positive urine and the metabolome of those specimens. While we were able to make molecular assignments for the dominant spectral differences (e.g., collagen, DNA, phosphatidylinositol, and others mentioned in the Results and S1 File), we note that several more minor contributors exist and likely are significant as well. Examples of these might include biomarkers such as NMP-22 and bladder tumor associated antigens present in bladder cancer urine [51]. However, since Rametrix^TM^ relies on chemometrics (i.e., extracting information from Raman spectra representing many molecules), individual biomarkers do not need to be identified specifically in a specimen to build an effective urine screen for BCA. The analysis specifically detects broad metabolomic signatures of disease. Thus, it is a combination of many molecular factors (some unknown at this point) that cause BCA-positive urine to be distinctly different. Nonetheless, we have begun the process of relating these spectral differences to specific metabolites and patterns using Raman spectral reference libraries, and we hypothesize this may result in a new set of metabolomic biomarkers (i.e., molecular signature) for BCA. However, more research is needed to validate these initial spectral findings with more patient samples, use of several analytical standards, and exploration of the minor components contributing to the molecular signature variances. We anticipate that no particular molecule, or small subset, will be indicative of BCA alone. It is likely that the entire molecular signature, analyzed using Rametrix^TM^, will be necessary to relate the urine metabolome to the presence of BCA. The leave-one-out analysis with Rametrix^TM^ PRO determines how well DAPC models will perform in characterizing new urine specimens. The results of the Rametrix^TM^ model to identify BCA-positive and BCA-negative patients of the Urology Clinic Dataset (Table 3 with 22 PCs) are used here to further illustrate the concepts of sensitivity, specificity, PPV, and NPV. Of those in a patient population, all who test BCA-positive by the Gold Standard (i.e., diagnostic tests and physical exam), 82.4% of these would test positive with the Rametrix^TM^ screening test (sensitivity). In this population, of those who test negative by the Gold Standard, 79.5% of these people would test negative with the Rametrix^TM^ screen (specificity). Of all who test positive with the Rametrix^TM^ screen, 63.6% of these would test positive with the Gold Standard (PPV). Finally, of all to test negative with the Rametrix^TM^ screen, 91.2% of these would also test negative with the Gold Standard (NPV). We certainly acknowledge that these values of sensitivity, specificity, PPV, and NPV still fall short of clinical relevance, and this demands further study. However, given the limited samples size (i.e., 56 specimens in the Urology Clinic patient dataset), we believe this study serves as a valid proof-of-concept. Since the sensitivity, specificity, PPV, and NPV all exceed the random chance value (50%) for a BCA-positive or BCA-negative designation, we believe there is justification for larger clinical studies of BCA patients and those with other GU pathologies, which will improve these metrics of this proposed urine screen.

We also recognize that the DAPC model architecture can be altered by including different numbers of PCs, and that better-performing models were chosen in this study based on performance. In future studies with larger patient populations, we expect different model architectures to emerge as optimum until a clinically-relevant urine screen is established. Once used clinically, the model architecture will remain static. In this study, we showed that by tuning the number of PCs used to build the model, higher sensitivity or specificity of the Rametrix^TM^ screening test could be achieved. High sensitivity would ensure more true-positive BCA cases are identified and recommended for further Gold Standard (i.e., definitive diagnostic) testing. Likewise, maximizing PPV may be useful in that those who screen positive have a higher likelihood of also testing positive by the Gold Standard. Given the invasive nature and resources required for Gold Standard testing for BCA, high PPV is a desirable attribute for the screening test. This also favors maximizing specificity (i.e., the true-negative rate) to minimize the false-positive rate. Those falsely identified by the screen as BCA-positive would, of necessity, undergo Gold Standard testing (which would occur if the Rametrix^TM^ screen did not exist) and be re-classified as BCA-negative with the results of those tests and exams. Extended clinical testing and thorough analysis of the costs/benefits will enable proper tuning of Rametrix^TM^ models and metrics to better align with clinical goals. Of course, the option exists to build multiple Rametrix^TM^ models with each tuned to favor different metrics. Further Gold Standard testing could be designed in response to which, or how many, of the Rametrix^TM^ models return a BCA-positive result. In addition, further developments could include “At Risk” predictions in addition to “BCA-positive” and “BCA-negative.” This new “At Risk” classification would arise from the region(s) of overlap in DAPC plots of our training datasets. These are apparent in Figs 3, 5 and 6 in this study. Of course, larger clinical trials will be needed for all of the scenarios discussed above to be considered.

In addition to these observations, we have noted the predictive power of DAPC models is influenced by the size of the spectral dataset used to build the model. Including more spectra generally improves predictive performance, but the number of positive and negative samples should be kept relatively balanced. Care should also be taken to ensure that even the negative samples are representative of the setting in which the predictions will be performed. Using negative samples that include many potentially obfuscating factors (e.g., hematuria and pyuria associated with infectious cystitis) will reduce estimated sensitivity, but more accurately portrays the true predictive power under worst case scenarios. If, however, Rametrix^TM^ is used in combination with other techniques or known patient history, the range of applicable negative training data could be reduced, resulting in improved screen metrics.

In this study, for example, the Healthy Volunteers dataset was largely composed of healthy college student volunteers (median age = 22); whereas, the Urology Clinic Patients dataset had a much higher median age (62 years) and included a significant portion of individuals with other GU conditions. If one were to design a screening strategy for cancer recurrence, the youthful healthy individuals may not be a representative source of training data. A recurrence screen scenario was tested in this study with the BCA-positive spectra compared to patients with BCA in remission, but predictions did not exceed random chance, largely due to the low sample size of patients with remission. More patients in these populations are also needed in extended clinical trials.

With large enough datasets, Rametrix^TM^ showed the technology was capable of distinguishing between different kinds of diseases in this study. Not only can it distinguish BCA and ESKD, but it revealed the capability to distinguish among different GU conditions. However, these distinctions had lower predictive power, owing largely, again, to the limited population size and wide variety of conditions aggregated in the category. This was also true of establishing separate screens based on sex in this study, where the low population size was the limiting factor. In a clinical setting, however, these factors will be important and should be incorporated into a single urine screen or require the use of separate screens, based on specific patient identifiers. It will be imperative in extended clinical trials to expand upon these observations and consider the population size and composition carefully to include conditions that may influence urine composition.

With sufficiently-sized spectral datasets, Rametrix^TM^ provides an attractive method for BCA screening. More definitive diagnosis always relies on urine cytology and cystoscopy, but Rametrix^TM^ boasts several advantages as a screening method. Urinalysis by Rametrix^TM^ is significantly less invasive than cystoscopy, does not require a trained pathologist, requires relatively inexpensive equipment and supplies, and results can be obtained remotely by technicians. We envision Rametrix^TM^ also being used for early and routine screening of individuals at high risk for developing BCA such as heavy smokers [52] or plastics factory workers [53]. Rametrix^TM^ has potential use for screening treated BCA patients for signs of recurrence. We intend to expand on the results of this study with samples obtained from ongoing clinical studies.

## Supporting information

S1 File (XLSX)
